# Cocaine-Induced Pituitary and Subdural Brain Abscesses and the Treatment Challenges

**DOI:** 10.7759/cureus.20821

**Published:** 2021-12-30

**Authors:** Mansoor Zafar, Samuel Vaughan, Bao Khuu, Sophiya Shrestha, Elisabetta Porruvecchio, Abubakar Hadid

**Affiliations:** 1 Gastroenterology and Hepatology, Conquest Hospital, East Sussex Healthcare NHS Trust, St. Leonards-on-Sea, GBR; 2 Radiology St4, Conquest Hospital, St. Leonards-on-Sea, GBR; 3 General Internal Medicine, Conquest Hospital, East Sussex Healthcare NHS Trust, St. Leonards-on-Sea, GBR; 4 Internal Medicine, Conquest Hospital, East Sussex Healthcare NHS Trust, St. Leonards-on-Sea, GBR

**Keywords:** patient education, mri, illicit drugs, hypopituitarism, cocaine

## Abstract

Cocaine is a well-known recreational drug with stimulant effects associated with relevant social, economic, and clinical implications. The most common route of abuse is via snorting. It has high addictive potential. Furthermore, one of the most well-known symptoms of a chronic user is chronic rhinitis. In the medical journals, there are numerous reports of complications, including lesions affecting the nasal septum, nasal sinuses, and even brain abscesses. We came across a 41-year-old male patient with severe manifestations of chronic cocaine use involving nasal, paranasal, and visual symptoms and signs. However, the most devastating was the complication of pituitary abscess, as a sequela to chronic cocaine sniffing. This case highlights the clinical, diagnostic, and management challenges with a multi-disciplinary approach. Last but not least, was the role of patient counselling and education. This ensured compliance towards management with a favourable outcome, which was rewarding for both the patient and the medical team involved in the care of the patient. It would hopefully create more awareness and assist in abstinence. We also hope it would incite more effort towards data collection and analysis, as well as allow us to explore the actual incidence of its use and devastating complications, which to date, for reasons of disguise and denial, remain somewhat ambiguous.

## Introduction

The incidence of brain abscesses in the developed world has been reported to be approximately 1500-2500 cases per year; this number is greater in the developing countries [1]. Sinusitis, with contiguous and haematological spread, has been linked with the formation of brain abscesses [2]. A computer tomogram (CT) scan is a rapid way of quantifying the abscesses in the brain. However, magnetic resonance imaging (MRI) can help to differentiate between brain tumours or cysts and brain abscesses [2]. Immunocompromised states, endocarditis, and lung empyema are all risk factors [2]. Headaches, confusion, and fever are common presentation symptoms [2,3]. Cocaine use with sinusitis, followed by frontal lobe abscess, has been previously reported in literature, and it has been managed with craniotomy [3]. We are presenting a complex case with involvement of the nasal septum, sinuses, subdural and even the pituitary gland, with related diplopia, scotoma and panhypopituitarism, which posed a clinical challenge towards successful management.

## Case presentation

A 41-year-old male, referred from the emergency department at the district general hospital to the on-call medical team, presented with a history of generalised headaches and collapsed with a complaint of being unable to weight bear and feeling exhausted, weak, and feverish. He recently moved to the area, with no past surgical nor medical history of any ailments recorded or stated, and he was not on any regular medications. His social history included being a non-smoker, non-alcoholic, working as manual labour and living with his wife and two kids. He gave a history of being a non-smoker and only occasionally drinking alcohol. Initial observations were a heart rate of 100 beats per minute and sinus rhythm on ECG. His blood pressure was 100/70 mmHg, his respiratory rate was 19/min, his oxygen saturation was 97% on air, and his temperature was 38 °C. On cardiovascular examination, he did not demonstrate any murmurs, and his urine dip-stick was negative for blood and had no other stigmata of infective endocarditis. On neurological examination, he was found to have diplopia secondary to left partial third nerve palsy and upper division fifth cranial nerve palsy. A slit-lamp examination revealed bilateral optic neuropathy, causing bilateral central scotoma. Blood tests showed C-reactive protein (CRP) of 101 mg/L (0-5), white cell count (WCC) of 13.6 × 109/L (4-11), and neutrophils of 8.76 × 109/L (2-7.5). The vascular screening, including anti-nuclear antibodies (ANA) and anti-neutrophilic cytoplasmic antibodies (ANCA), was negative. His respiratory examination and chest X-ray were inconclusive. His 12-lead ECG showed only sinus tachycardia. Echocardiograms, both transthoracic (TTE) and transoesophageal (TOE), did not demonstrate any vegetation.

**Figure 1:**
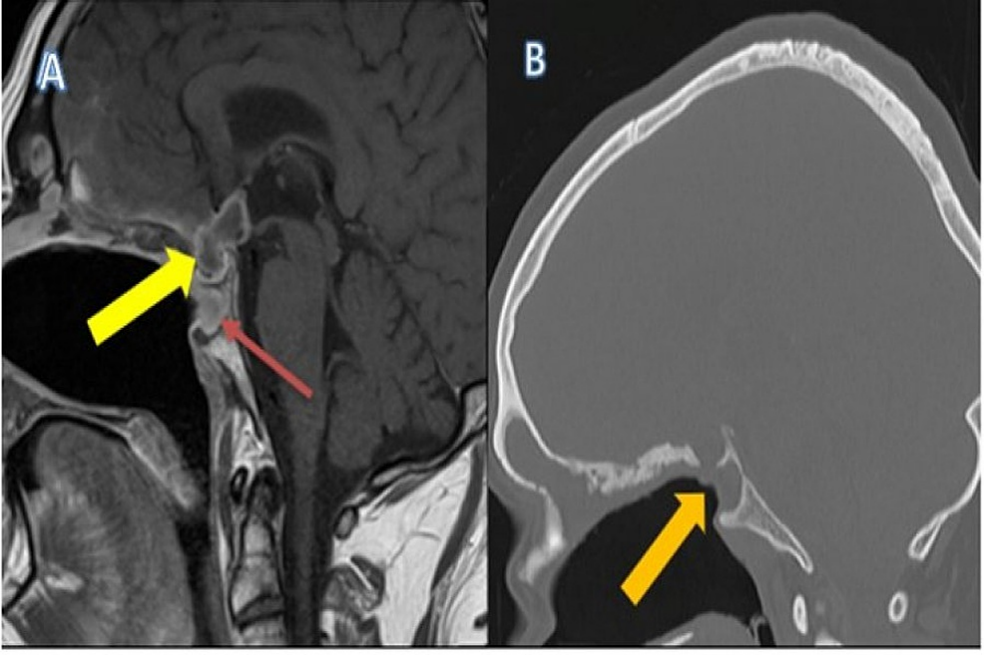
(A) MRI, sagittal T1 post contrast, demonstrating peripherally enhancing lesion arising from the pituitary fossa (yellow arrow). Enhancing soft tissue is seen in the sphenoid sinus region, adjacent to the pituitary fossa. Erosion of the Sella turcica allowing communication between the pituitary fossa and sphenoid sinus region (red arrow). (B) Right image: CT, sagittal non-contrast, demonstrating complete bony erosion of the entire septum causing loss of distinction between the paranasal sinuses (orange arrow).

An acute non-contrast CT head was performed which demonstrated a pituitary fossa lesion with erosion of the Sella turcica, allowing communication to the sphenoid sinus and extensive bilateral frontal lobe oedema. Of note, the entire nasal septum was absent, causing loss of the distinction between the paranasal sinuses. The overall appearance indicated a destructive rather than congenital or iatrogenic cause. A MRI was performed in due course, indicating the pituitary mass was most likely an abscess, compressing upon the optic chiasma. An abnormal signal was seen extending into the cavernous sinuses with encasement of the cavernous portions of the ICAs bilaterally. There was shallow frontal subdural empyema with associated leptomeningeal enhancement and frontal lobe oedema, indicating meningitis (Figure 1).

Meanwhile, the blood culture demonstrated no growth after five days. A nasal swab suggested methicillin-resistant *Staphylococcus aureus* (MRSA) with cultural sensitivity to Mupirocin and resistance to Flucloxacillin. Cerebral spinal fluid analysis demonstrated WBC <5 × 106/L and no growth demonstrated in culture. Serology for HIV, hepatitis B virus and hepatitis C virus was negative.

Consequentially, a drug use history was obtained from the patient, in which he admitted to a long history of cocaine use. A diagnosis of cocaine-induced bi-frontal and pituitary abscesses with panhypopituitarism was made. Following this, he was referred to neurosurgery at the tertiary centre (University Teaching Hospital) with successful drainage of supra-sellar collection via trans-sphenoidal approach. Postoperatively, the samples grew *S. aureus*. His case was discussed with the endocrinology and microbiology teams, and he was managed with linezolid and meropenem based on cultural sensitivity and intravenous glucocorticoids. Unfortunately, he self-discharged from the tertiary centre the very next day, against medical advice. He agreed to take oral antibiotics, glucocorticoids, and mineralocorticoids.

**Figure 2:**
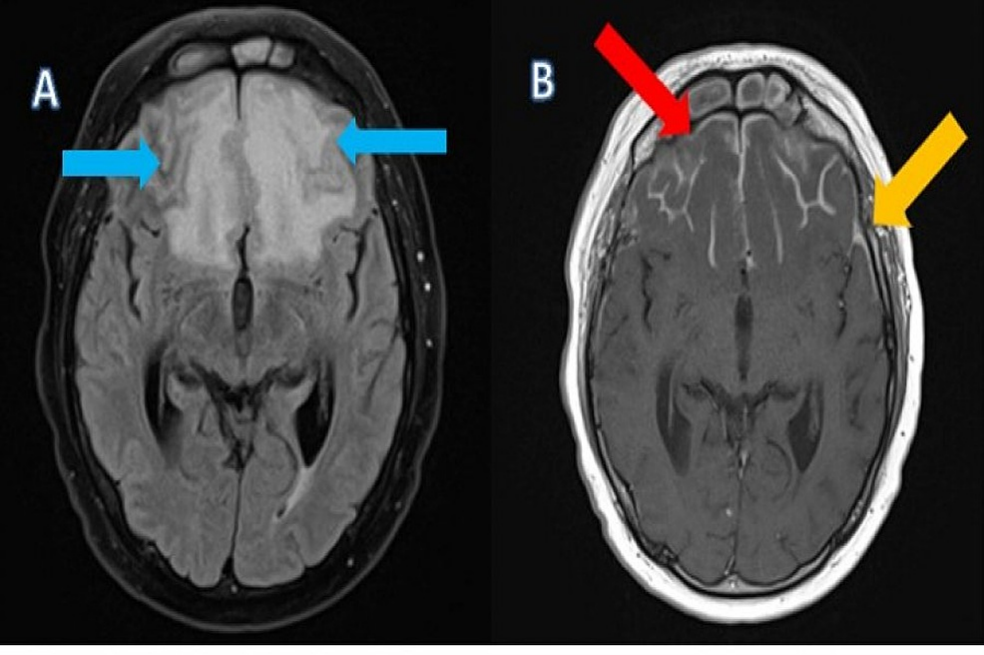
(A) MRI, axial T2 FLAIR demonstrating extensive oedema in the frontal lobes bilaterally (blue arrows). (B) MRI, axial T1 post-contrast, demonstrating leptomeningeal enhancement overlying the frontal lobe gyri (red arrow) and extending to the temporal lobes (orange arrow).

Three months later, he reported to the district general hospital again with a complaint of headaches. His repeated CT followed by MRI head scans showed significant resolution of pituitary collection. However, it showed increased signal strength bilaterally in frontal, temporal, and midline paracalcine areas (Figure 2).

He was started on intravenous vancomycin and meropenem in consultation with microbiology. However, he developed an urticarial rash with facial flushing. An impression of vancomycin related side-effects was made. The patient wished to be discharged. On further inquiries, he mentioned feelings of hopelessness towards recovery and persistent challenges like the vancomycin-associated rash and his questions towards any realistic improvement, if any at all. He was explained all the pros and cons of his condition and treatment plans, resulting in an agreement to stay as an in-patient to get optimised treatment towards resolution of his symptoms. In agreement with microbiology and neurosurgery, his antibiotics were changed to intravenous meropenem and linezolid for three months while inpatient, with a conservative approach advised by the neurosurgery team. In consultation with the endocrinology team, he was continued on oral hydrocortisone and fludrocortisone with advice to double the steroid dose for sick days. His diplopia resolved, and he was advised strict abstinence of cocaine use in the future, to which he agreed. 

## Discussion

Cocaine use for recreational purposes has been known world-wide, across all time periods and through the ages. The Office for National Statistics reported scary statistics for the year ending in 2020 across England and Wales for powder cocaine use. They revealed 873,000 people (within the age group of 16 to 59) with reported use of this drug in the year 2020 (2.6% of the population) [4].

The United Kingdom, Spain, and Italy have been the countries with the highest adult consumption rates and where the burden of dependency has deeper effects on the population [5]. However, this remains challenging due to the fact that patients avoid getting checked and attempt to hide or blatantly deny the use and addiction [5]. Hence, the actual incidence of cocaine abuse and its complications, in actuality, remains unknown. Perhaps, it would be greater than what could be recorded. The occurrence of nasal septal involvement [6], oral involvement [7], and brain abscesses, particularly frontal lobe abscesses [8], have all been reported numerous times.

The cocaine-induced panhypopituitarism, both with positive and negative ANCA, has been reported in the literature [9]. Insel and Dhanjal have previously reported the first such case of pituitary involvement with erosions of empty sella-turcica and diabetes insipidus managed with a multi-disciplinary approach [10]. Palatal perforations with complex management by ENT surgeons have been reported with favourable outcomes [11].

However, we report here the case of a patient with complex issues regarding his fears and feelings of hopelessness. This led to a self-discharge, followed by a second attempt at self-discharge, making it a challenging case to manage. This case highlights the importance of educating a patient on their condition and coming to an agreement for them to remain in hospital to optimise treatments for their overall recovery and well-being. It would be interesting to see more data collection and analysis towards actual incidence, which would assist with more awareness and the hopeful outcome of more influence towards abstinence.

## Conclusions

The true incidence of cocaine-induced complications remains unknown. This may probably be even far greater than what has been reported so far. A need for large-scale data collection and analysis of complexities is warranted. More awareness of strong addiction potential along with devastating complications is needed to improve abstinence and increase awareness. A strong support system, including counselling and rehabilitation, would encourage persistent abstinence.

The physicians and the medical students need to be aware of the treatment challenges involving complex situations, including patients’ fears and lack of faith in the steps towards recovery. Patients’ education and discussion towards pros and cons and the overall management plan would increase compliance with a favourable outcome, as in our case.
